# Generation and Characterization of a Novel Recombinant Antibody against LMP1-TES1 of Epstein-Barr Virus Isolated by Phage Display

**DOI:** 10.3390/v5041131

**Published:** 2013-04-22

**Authors:** Dawei Zhang, Yuan Mao, Qing Cao, Lin Xiong, Juan Wen, Renjie Chen, Jin Zhu

**Affiliations:** 1 Department of Otolaryngology Head and Neck Surgery, The Second Affiliated Hospital of Nanjing Medical University, 121 Jiangjiayuan, Nanjing 210011, China; E-Mails: chinarenzdw@yahoo.com.cn (D.Z.); ufo-43@163.com (Q.C.); wj20091555@163.com (J.W.); 2 Huadong Medical Institute of Biotechniques, 293 Zhongshandong Road, Nanjing 210002, China; 3 Department of Otolaryngology Head and Neck Surgery, Jiangsu Province Official Hospital, 65 Jiangsu Road, 210029 Nanjing, China; E-Mail: ymaoent@yahoo.com; 4 Department of Pathology, The Second Affiliated Hospital of Nanjing Medical University, 121 Jiangjiayuan, Nanjing 210011, China; E-Mail: dongliju0322@yahoo.com.cn

**Keywords:** EBV, LMP1, phage antibody library, nasopharyngeal carcinoma, Fab antibody

## Abstract

Latent Membrane Protein 1 (LMP1) is a primary target for controlling tumorigenesis in Epstein-Barr virus related malignancies; in this study, we aimed to develop a specific antibody against the TES1 domain of the oncogenic LMP1. We screened a full human naïve Fab phage library against TES1 peptide, which consisted of C terminal-activating regions proximal 44 amino acids. After three rounds of panning, enrichment and testing by phage ELISA and further analyzed by DNA sequencing, we selected a phage clone with the highest affinity to LMP1-TES1 and designated it as htesFab. The positive clone was expressed in *Escherichia coli* and the purified htesFab was characterized for its binding specificity and affinity to LMP1. ELISA, immunofluorescence and FACS analysis confirmed that htesFab could recognize LMP1 TES1 both *in vitro* and in LMP1 expressing HNE2-LMP1 cells. Furthermore, MTT assay showed that htesFab inhibited the proliferation of HNE2-LMP1 cells in a dose-dependent manner. In summary, this study reported the isolation and characterization of human Fab, which specifically targets the C terminal region/TES1 of LMP1, and has potential to be developed as novel tool for the diagnosis and therapy of Epstein-Barr virus related carcinoma.

## 1. Introduction

Epstein-Barr virus (EBV) is present in virtually poorly and undifferentiated nonkeratinizing nasopharyngeal cancer (NPC) regardless of geographic origin, and the viral antigens expressed by the tumor provide potential target antigens for immunotherapy [1,2]. Latent Membrane Protein 1 (LMP1) is considered as a major EBV oncoprotein [3]. LMP1 positive NPCs are more progressive than LMP1 negative NPCs and show increased tendency of lymph node metastasis [4]. Structurally, LMP1 is an integral membrane protein consisting of a short cytoplasmic N-terminus of 20 amino acids, a trans-membrane domain with six membrane-spanning segments that anchor LMP1 in a patchy distribution along the plasma membrane, and a long cytoplasmic C-terminus of 200 amino acids [5,6]. LMP1 has two C-terminal cytosolic domains, transformation effector sites (TES)1 and -2, which resemble the tumor necrosis factor (TNF) receptor and modulates several cellular signaling pathways [7,8]. Two domains TES1 and TES2 have been identified within the C-terminus of LMP1 as being important for B-lymphocyte growth transformation and phenotypic changes in a variety of cell types. TES1 (transformation effector site 1) comprises the membrane-most proximal 34 amino acids (amino acid residues 187–231) and contains aP204×Q206×T208D209 motif, which serves as a docking site for adapter proteins of TNF receptor (TNFR)-associated factor (TRAF) family such as TRAF1, TRAF2, TRAF3, and TRAF5. TES1 is sufficient for mediating initial B-lymphocyte growth transformation [9,10]. The TES1 amino acid sequence is similar to that of CD40 and CD30 which is critical for NF-kB activation mediated by TRAF1, TRAF2 and TRAF5 [10,11,12,13,14,15]. 

Because LMP1 is a primary target for controlling tumorigenesis in EBV-related malignancies, in this study we aimed to develop a specific antibody against the TES1 domain of the oncogenic LMP1. We screened a full human naïve Fab phage library against TES1 peptide(pLMP1-TES1), which consisted of C terminal-activating regions proximal 44 amino acids. We selected a human anti-LMP1 TES1 antibody Fab (htesFab) and characterized its binding specificity and affinity to LMP1.

## 2. Results and Discussion

### 2.1. Results

#### 2.1.1. Selection of Specific LMP1 Binding Phage and Nucleic Acid Analysis of htesFab Clones

**Figure 1:**
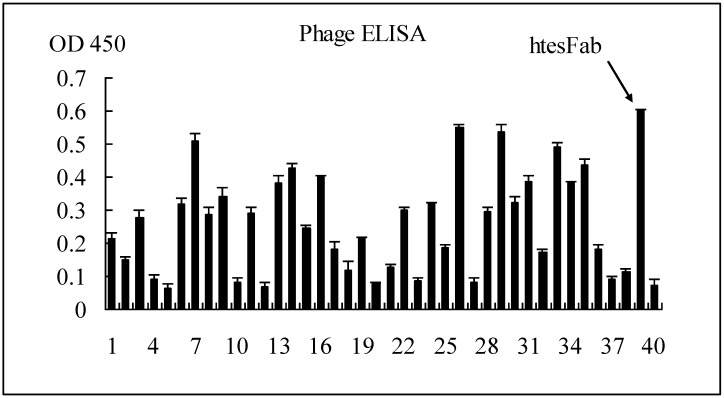
ELISA results of 40 individual phage clones randomly picked up from the eluted phage pool after the 3rd round of bio-panning. Purified human pLMP1-TES1 was coated at 400 ng per well, and 50 μL supernatant of each phage was added to each well for ELISA.

After three rounds of panning, 40 single phage clones were randomly picked up. The output/input increased gradually from 1.0 × 10^−7^ to 1.6 × 10^−5^, showing the continuous enrichment of anti-LMP1-TES1 Fab clones (Table 1). The specific binding to pLMP1-TES1 by Fab was tested by phage ELISA. The results showed that several positive clones were selected (Figure 1). One of the positive clones with the highest OD value was named htesFab and analyzed by DNA sequencing. The amino acid sequences of VL and VH of htesFab were shown in Table 2. The htesFab H and L sequences (VH and V-KAPPA domain sequences, respectively) were automatically analyzed with IMGT/V-QUEST software, which identifies the immunoglobulin germ line V, (D), and J genes from which a specific immunoglobulin chain is derived. For the htesFab H VH domain (V-D-J-REGION), the IGHV gene clearly belongs to the IGHV3 subgroup. The V-REGION was recognized as originating from a IGHV gene similar to the human germ line Homsap IGHV3-30*04 F allele, with a 99.31% nucleotide sequence identity in the V-REGION frameworks. The H J-REGION gene was recognized by the IMGT/V-QUEST tool as originating from a htesFab IGHJ gene close to the human germ line IGHJ4*02 gene. The D-REGION was recognized as originating from a IGHD gene similar to the human germ line IGHD6-6*01 gene. For the V-KAPPA (V-J-REGION) domain, the htesFab L V-REGION was recognized as originating from a IGKV gene similar to the human germ line Homsap IGKV1-8*01 F, with a 98.57% nucleotide sequence identity in the V-REGION frameworks. The J-REGION gene was recognized by IMGT/V-QUEST as originating from a IGKJ gene similar to the Homsap IGKJ4*01 F human germ line gene.

**Table 1:** Selective enrichment of Fabs from Fab library during panning.

Fab library	1st round	2nd round	3rd round
Phage input (cfu)	2.0 × 10^12^	2.0 × 10^10^	1.0 × 10^10^
Phage output (cfu)	2.0 × 10^5^	2.5 × 10^5^	1.0 × 10^5^
Output/input	1.0 × 10^−7^	1.25 × 10^−5^	1.6 × 10^−5^

**Table 2:** Amino acid sequences of VL and VH genes of htesFab clone.

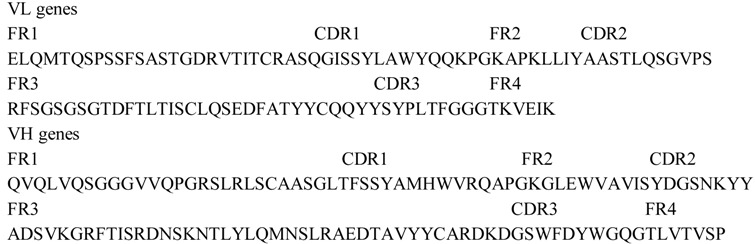

#### 2.1.2. Expression and Purification of htesFab

**Figure 2:**
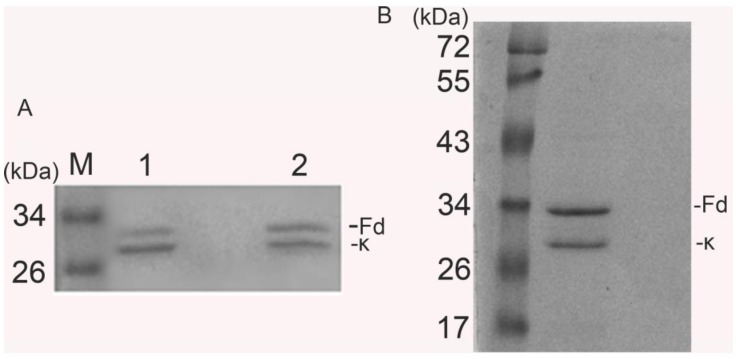
Western blotting characterization of htesFab fragment expressed in *E. coli*. Lane 1: Top10F' cell lysate control; lane 2: 25 mL of cell lysates after sonication. Mouse antihuman Fab HRP conjugate was used at 1:1,000 dilution (**A**). Purified htesFab fragment was separated on a 10% SDS-PAGE gel and stained with Coomassie blue. The double bands were heavy chain Fd (top) and light chain k (bottom) (**B**).

Soluble expression of htesFab was induced overnight at low temperature (25 °C). htesFab was expressed mainly in the periplasmic space of *E. coli* (Figure 2A). SDS-PAGE and Coomassie Blue staining showed equal expression of heavy and light chains. The purity was above 95% after Protein L affinity purification (Figure 2B). 

2.1.3. htesFab Binds the TES1 Domain of LMP1

**Figure 3:**
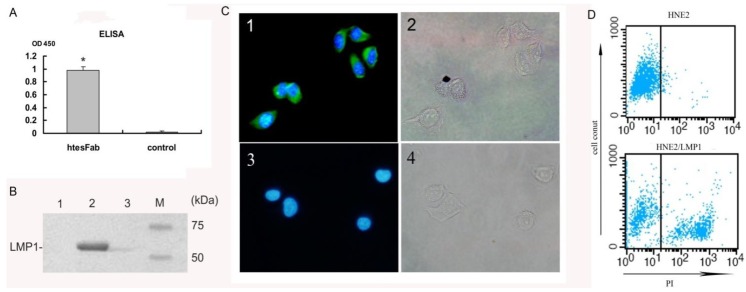
Characterization of Fab binding with LMP1-TES1 domain. (**A**) ELISA showed that htesFab bound LMP1-TES domain in native confirmation (*p* < 0.05); (**B**) Immunoprecipitation analysis for the detection of LMP1 protein. LMP1 was 53 kDa; Line 1: HNE2 cells; line 2: HNE2-LMP1 cells; line 3: unrelated Fab fragment (**C**); Immunofluorescence analysis showed that htesFab labeled LMP1 in the intracellular and plasma membranes in HNE2-LMP1 cells (green), cell nuclei were stained with DAPI (blue) (×200, Olympus digital camera); (**D**) FACS analysis showed the binding of htesFab to HNE2-LMP1(40.35%) and HNE2 cells(4.15%) (blue dots). Background staining was obtained by PBS.

htesFab immunoreactivity was tested by its ability to bind the TES1 domain of LMP1. ELISA assay showed that the mean value OD450 nm of htesFab was 0.918 ± 0.056 compared to the negative control (0.022 ± 0.015) (*p* < 0.05) (Figure 3A), confirming that the purified htesFab recognized pLMP1-TES1. Immunoprecipitation analysis showed that approximately 53 kDa LMP1 protein was detected in HNE2-LMP1 cells but not in HNE2 cells (Figure 3B). Next, we performed immunofluorescence analysis with htesFab to visualize the TES1 antigen in HNE2-LMP1 cells. The results showed that htesFab labeled the antigen (green) in the intracellular and plasma membranes in HNE2-LMP1 cells, but not in HNE2 cells (Figure 3C). Cell nuclei were stained blue with DAPI. Furthermore, FACS analysis showed that htesFab bound with much higher affinity to HNE2-LMP1 cells than to HNE2 cells (Figure 3D). Taken together, these data demonstrate that htesFab binds the TES1 domain of LMP1 with high specificity and affinity.

2.1.4. htesFab Inhibits the Proliferation of HNE2-LMP1 Cells *in Vitro*

**Figure 4:**
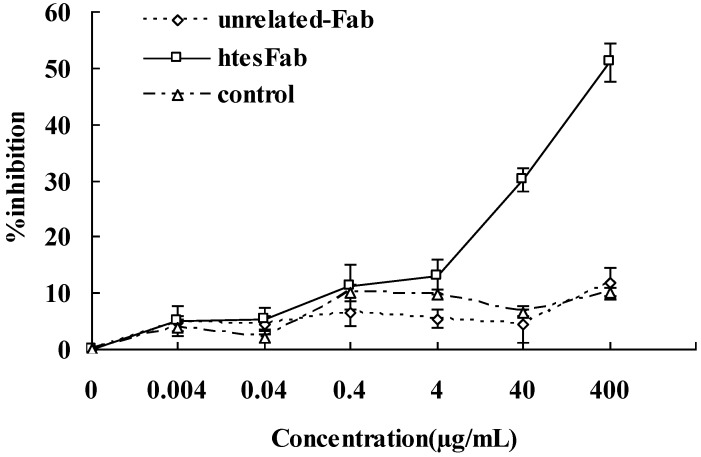
htesFab inhibits the proliferation of HNE2-LMP1 cells *in vitro*. The cells were treated as indicated and harvested 48 h later. The proliferation of cells was assessed by MTT assay to calculate the proliferation inhibition rate (%).

To determine the efficacy of htesFab to specifically inhibit the growth of HNE2-LMP1 cells *in vitro*, HNE2-LMP1 cells were treated with different concentrations of htesFab or unrelated Fab as control. MTT assay showed that htesFab inhibited cell proliferation in a dose-dependent manner. At the concentration of 0–400 μg/mL, htesFab was able to inhibit the proliferation of HNE2-LMP1 cells significantly, compared with unrelated Fab treated cells or untreated cells (Figure 4).

### 2.2. Discussion

The leading cause for therapeutic failure in patients is an advanced stage of the disease. Therefore, antibody application is essential for diagnosis of NPC at early stages and new biologic therapies of NPC. Elevated Rta, VCA, EA, NA-1 antibody titers targeting EBV antigens have been used as screening or diagnostic biomarkers for NPC [16,17,18,19,20]. Based on the results of the present study, the combined measurement of the anti-Rta and anti-EA antibodies could increase the sensitivity and the validity for the serologic screening and diagnosis of nasopharyngeal carcinoma [21]. Additionally, new treatment options including targeted monoclonal antibodies for example Cetuximab, a monoclonal antibody that inhibits the epidermal growth factor receptor (EGFR), is being studied in NPC. Bevacizumab, a monoclonal antibody that inhibits VEGF, is currently being investigated with cisplatin based chemo-RT in a Phase II trial [22,23] These emerging clinical studies are beginning to define their role.

In this study, from a large phage-display library of naïve human Fab fragments we successfully selected human Fab fragment against TES1, a well-characterized tumor antigen with potential applications in clinical diagnosis and therapy. The selected antibody fragment, designated htesFab, was isolated and its specificity was confirmed by ELISA, immunofluorescence, and FACS analysis. In addition, we demonstrated that htesFab inhibited the proliferation of HNE2-LMP1 cells *in vitro*.

The LMP1 C terminal region is involved in several cell signaling pathways related to apoptosis, cell proliferation, and invasion. LMP1 TES1 could upregulate several signaling pathways, including NF-κB, MAPK, JNK/AP-1 and PI3K [5]. We isolated fully human Fab phage after panning against a transformation effector site 1 (TES1) peptide, which comprises the 187 to 231 amino acid residues of the cytoplasmic tail of LMP1 and serves as a docking site for adapter proteins of TRAF family. Using this strategy, we demonstrate that the htesFab could bind LMP1 TES1 peptide. 

Targeting of LMP1 TES1 has become one of the most useful treatment modalities for refractory NPC. Consequently, phage display technique and gene engineering technology have been utilized for the development of scFv against LMP1 TES1 [24,25,26]. Nevertheless, only a few scFv antibodies have been demonstrated to be effective in modulating target proteins functions *in vivo* [27,28,29,30]. The major problems include incorrect folding of the scFv due to inefficient disulfide bond formation, which leads to low expression or short half-life of the scFv [31]. Although Fabs in general are more difficult to assemble, more likely to be degraded, have lower yields as soluble fragments compared with scFvs, Fabs have no dimerization problem and tend to be more stable.

In our study, we were able to generate a human Fab fragment that bound LMP1 TES1 domain. The repeated panning with coated pLMP1-TES1 in microliter plates ensured the enrichment of specific LMP1 TES1 binding phages. After three rounds of panning, we selected one of positive clones with the highest OD value in ELISA and named it as htesFab. ELISA, immunofluorescence and FACS analysis confirmed that htesFab could recognize LMP1 TES1 both *in vitro* and in LMP1 expressing cells (HNE2-LMP1 cells). Furthermore, we found that htesFab inhibited the proliferation of HNE2-LMP1 cells in a dose-dependent manner.

## 3. Experimental

### 3.1. Phage Library, Helper Phage and Bacterial Strains

A human naive Fab phage library was constructed as previously described [32]. Before the first-round panning, the library was titrated and 2 × 10^12^ phage clones were collected for panning. The VCSM 13 helper phage and the *E. coli* strain *Xl1.blue* and another *E. coli* strain, Top 10 F', were provided by Key Laboratory of Antibody Technique of Health Ministry, Nanjing Medical University. Both strains were tested to exclude any wild-type phage contaminations. 

### 3.2. Cell Lines and Peptides

Two cell lines were used for biopanning and Fab characterization as well as *in vitro* bioassays: human nasopharyngeal carcinoma cell line HNE2 (LMP1 negative) and human nasopharyngeal carcinoma cell line HNE2-LMP1 (LMP1 positive). They were purchased from XiangYa Central Experiment Laboratory (Hunan, China) and cultured in RPMI-1640 medium (GIBCO® Invitrogen) supplemented with 10% fetal bovine serum (FBS). A biotinylated 145aa peptide (H-G-Q-R-H-S-D-E-H-H-H-D-D-S-L-P-H-P-Q-Q-A-T-D-D-S-G-H-E-S-D-S-N-S-N-E-G-R-H-H-L-L-V-S-G-K-G-G-G-G-S-H-G-Q-R-H-S-D-E-H-H-H-D-D-S-L-P-H-P-Q-Q-A-T-D-D-S-G-H-E-S-D-S-N-S-N-E-G-R-H-H-L-L-V-S-G-K-G-G-G-G-S-H-G-Q-R-H-S-D-E-H-H-H-D-D-S-L-P-H-P-Q-Q-A-T-D-D-S-G-H-E-S-D-S-N-S-N-E-G-R-H-H-L-L-V-S-G-K) (TES1- G-G-G-G-S- TES1- G-G-G-G-S- TES1 polypeptide) corresponding to amino acid residues 187–231 of pLMP1-TES1 was synthesized by Saibaisheng Gene Technology Co., Ltd. (Shanghai, China).

### 3.3. Bio-Panning

Library screening was performed using the *naïve* human Fab phage display library. Antigen pLMP1-TES1was coated onto Maxisorb Immunotube (Corning brand) at 4 °C overnight. For panning, the pLMP1-TES1 polypeptide was added to the phage at concentration of 10 μg/mL (rounds 1), or 5 μg/mL (round 2, 3). The coated tubes/beads and the phage library were separately blocked in 5% MPBS (5% milk in PBS) for 1 h at room temperature (RT). Pre-blocked phage mixtures were then incubated with the coated tube/beads for 2 h at RT; unbound phages were eliminated by washing 10 times with PBS-T (0.05% Tween 20). Bound phages were treated with 2 mg/mL trypsin for 15 min at 37 °C and the eluted phages (phage output) were used to infect *Escherichia coli Xl1-blue* grown to OD600 0.6 and subsequently rescued with VCSM13 helper phage. The rescued phages were amplified and incubated at 37 °C overnight. After the phages, named phage input was purified by PEG precipitation, phage input as the phage library was used in next round. The same procedure was repeated for subsequent panning. It produced output 2,3 and input 2,3. 

### 3.4. ELISA Screening of LMP1-Binding-Positive Phage Clones

Single phage clones from the *E. coli Xl1-blue* infected by the 3rd round of eluted phage were picked up and grown in 1 mL SB medium containing 50 mg/mL carbenicillin and 1% glucose, with shaking at 37 °C until the exponential phase. VCSM13 helper phage (10^9^) was added to each vial. The culture was shaken overnight with 70 μg/mL kanamycin. Forty microliters of supernatant from each vial was added to each well of 96-well EIA plates coated with 400 ng pLMP1-TES1and blocked with a 5% milk blocking buffer (5% milk, 0.5% Tween-PBS). After incubation at room temperature for 1 h, the plates were washed with 0.5% Tween-PBS, and incubated with 50 μL of 1:4,000 diluted HRP conjugated anti M13 antibody (Amersham code number: 27-942-01) in blocking buffer for 1 h. The plates were washed again and incubated with 40 μL of HRP substrate solution (Pierce, Prod# 34021) for 30 min before stopping by the addition of 1 M H_2_SO_4_. The absorbance value at 450 nm was read by Multiskan Spectrum Microplate (Themo Instruments Inc., Waltham, MA, USA). 

### 3.5. Nucleic Acid Analysis of htesFab Clones

The sequences were analyzed online, using the International ImMunoGeneTics information system (IMGT) (data from the IMGT/LIGM database [33]) and compared with the sequences of the human germ line immunoglobulin genes using IMGT/V-QUEST.

### 3.6. Expression and Purification of a Soluble Fab Fragment

The recombinant Fab was expressed in *E. coli* Top 10 F strain. Briefly, the overnight culture of a single clone was incubated at 1:100 in SB medium with 50 mg/mL carbenicillin until the OD600 reached 1.0, then induced by 1 mM IPTG in the presence of 4% sucrose at 25 °C and harvested 24 h later. Western blotting was performed on both bacteria lysate and sonicated supernatant to determine the expression of Fab. The soluble Fab was purified from the periplasm of the bacteria by affinity purification using PROTEIN L kit (GenScript Corporation, Nanjing, China; Cat. No. L00239). Purified Fab was tested for the binding activity to pLMP1-TES1 using ELISA as described in the above. 

### 3.7. Immunoprecipitation

HNE2-LMP1 cells (1 × 10^6^) were lysed in RIPA buffer and the lysate was incubated with 50 μg/mL htesFab 1 mL and 100 μL Protein L-Agarose beads (GenScript Corporation, Cat. No. L00239) at 4 °C overnight. The beads were washed three times with 0.1% Tween-PBS and resuspended in 40 μL of 2 × SDS loading buffer, then boiled for 10 min. The samples were subjected to Western blot analysis to detect the precipitated LMP1, using LMP1 antibody (BD Pharmingen Cat. No. 559898) and HRP conjugated goat anti-mouse IgG at 1:500 dilution (BD Pharmingen). The HNE2 cells were used as negative control.

### 3.8. Immunofluorescence Assay

HNE2-LMP1 cells were grown on coverslips in 6-plates till 80% confluent. The coverslips were washed three times with ice cold PBS and fixed with 100% methanol at −20 °C for 5 min., blocked by 5% milk PBS at 37 °C for 1 h, and incubated with 50 μg/mL htesFab at 37 °C for 1 h followed by incubation with 1:50 FITC labeled anti human Fab IgG (Sigma, St. Louis, MO, USA; Cat: F5512) for 1 h in the dark. The cell nuclei were stained with 1 mg/mL HOECHST 33342 solution (1:10,000, Dojindo, Kumamoto, Japan) at 37 °C for 5 min, and the coverslips were mounted and observed by fluorescence microscopy. Cells showing strong green fluorescence were recorded as positive. The HNE2 cells were used as negative control. 

### 3.9. Fluorescence-Activated Cell Sorter Analysis (FACS)

Approximately 1 × 10^6^ cells of HNE2-LMP1 and 1 × 10^6^ HNE2 were washed three times with ice cold PBS, fixed with 100% methanol at −20 °C for 5 min, blocked by 5% milk PBS at 37 °C for 1 h, and incubated with 150 μL purified htesFab (20 μg/mL) at 4 °C for 12 h. After being washed with PBS, these cells were incubated with 100 μL of 1:50 diluted FITC labeled anti human Fab IgG (Sigma, Cat: F5512) for 30 min at 37 °C. The cells were washed and analyzed on the BD FACS Calibur using the Cell Quest program. Background staining was obtained by PBS at the same volume.

### 3.10. MTT Assay

HNE2/LMP1 cells were seeded in 96-well plates at a density of 5.0 × 10^3^ cells/well and cultured overnight, then fresh medium containing htesFab or unrelated antibody (0–400 μg/mL) was added to each well and incubated for 48 h. Subsequently, the culture medium was removed and 100 μL of MTT 3-(4,5-dimethylthiazol-2-yl)-2,5-diphenyltetrazoliumbromide) (1 mg/mL in RPMI 1640 ) was added to each well and incubated for 5 h at 37 °C in 5% CO_2_ incubator. Then the supernatant was removed, and 150 μL of dimethylsulfoxide (DMSO) was added to each well followed by shaking at 150 rpm for 5 min. Absorbance at 490 nm was determined spectrophotometrically. The cell growth inhibition rate (GIR) was calculated as follows: GIR = (1 − OD_490_ of treated cells/OD_490_ of untreated cells) × 100%. The cells without any antibody treatment served as the negative control. MTT assay was repeated at least three times.

## 4. Conclusions

In summary, this study reported the isolation and characterization of human Fab, which specifically targets the TES1 of LMP1. The Fab we generated may have great potential to be developed as novel tool for NPC diagnosis and therapy.
